# Respiratory neuropathology in spinocerebellar ataxia type 7

**DOI:** 10.1172/jci.insight.170444

**Published:** 2024-07-18

**Authors:** Debolina D. Biswas, Yihan Shi, Léa El Haddad, Ronit Sethi, Meredith Huston, Sean Kehoe, Evelyn R. Scarrow, Laura M. Strickland, Logan A. Pucci, Justin S. Dhindsa, Ani Hunanyan, Albert R. La Spada, Mai K. ElMallah

**Affiliations:** 1 Division of Pulmonary and Sleep Medicine, Department of Pediatrics, Duke University Medical Center, Durham, North Carolina, USA.; 2 Departments of Pathology and Laboratory Medicine, Neurology, Biological Chemistry, and Neurobiology and Behavior, and; 3 UCI Center for Neurotherapeutics, University of California Irvine, Irvine, California, USA.

**Keywords:** Neuroscience, Pulmonology, Expression profiling, Neurological disorders, Respiration

## Abstract

Spinocerebellar ataxia type 7 (SCA7) is an autosomal dominant neurological disorder caused by deleterious CAG repeat expansion in the coding region of the ataxin 7 gene (polyQ-ataxin-7). Infantile-onset SCA7 leads to severe clinical manifestation of respiratory distress, but the exact cause of respiratory impairment remains unclear. Using the infantile-SCA7 mouse model, the SCA7^266Q/5Q^ mouse, we examined the impact of pathological polyQ-ataxin-7 on hypoglossal (XII) and phrenic motor units. We identified the transcript profile of the medulla and cervical spinal cord and investigated the XII and phrenic nerves and the neuromuscular junctions in the diaphragm and tongue. SCA7^266Q/5Q^ astrocytes showed significant intranuclear inclusions of ataxin-7 in the XII and putative phrenic motor nuclei. Transcriptomic analysis revealed dysregulation of genes involved in amino acid and neurotransmitter transport and myelination. Additionally, SCA7^266Q/5Q^ mice demonstrated blunted efferent output of the XII nerve and demyelination in both XII and phrenic nerves. Finally, there was an increased number of neuromuscular junction clusters with higher expression of synaptic markers in SCA7^266Q/5Q^ mice compared with WT controls. These preclinical findings elucidate the underlying pathophysiology responsible for impaired glial cell function and death leading to dysphagia, aspiration, and respiratory failure in infantile SCA7.

## Abstract 2

Spinocerebellar ataxia type 7 (SCA7) is a hereditary neurological disease that causes problems with mobility and vision. Each generation of patients with SCA7 is more severely affected than their parents. Infants with SCA7 have problems with feeding, swal

## Introduction

Spinocerebellar ataxia type 7 (SCA7) is an autosomal dominant neurological disorder characterized by cerebellar ataxia, retinal dystrophy, dysarthria, dysphagia, aspiration, and respiratory dysfunction (1–6). SCA7 is caused by an expansion of CAG repeats in the coding region of the ataxin-7 gene (*ATX7*), which is translated into an expanded polyQ (polyglutamine) stretch in the ataxin-7 protein. Approximately 7 to 35 CAG repeats are present in normal *ATX7* (7–9). However, the symptoms of SCA7 manifest when there are 37 to more than 300 CAG repeats (4, 10–12). The number of CAG repeats is directly proportional to the severity of the symptoms and inversely proportional to the age of onset. Furthermore, SCA7 results in genetic anticipation — that is, the number of CAG repeats increases in consecutive generations and causes an earlier onset and more rapidly progressive severe disease (5, 13–16).

Infantile-onset SCA7 is associated with more than 200 CAG repeats and results in rapid disease progression and death within 1–3 years. Infants with SCA7 have severe pathology characterized by swallowing dysfunction and brainstem degeneration, and they often succumb to aspiration pneumonia and respiratory failure (2, 14, 17–19). We previously confirmed respiratory pathology in an infantile-SCA7 mouse model with severe disease and extensive CAG repeats — SCA7^266Q/5Q^-knockin mice (hereafter called SCA7 mice) carrying 266 CAG repeats (20). SCA7 mice have progressive respiratory dysfunction, with episodes of erratic breathing and prolonged apneic events. In addition, these mice have decreased phrenic and hypoglossal (XII) motor neurons (21). The phrenic nerves originate from the phrenic motor neurons in C3–C5 of the cervical spinal cord and innervate the diaphragm — the primary muscle of inspiration. Loss of phrenic motor neurons reduces respiratory capacity and leads to respiratory failure (22, 23). The XII nerves originate from the XII motor neurons in the medulla oblongata and innervate several extrinsic and intrinsic tongue muscles essential for motor function, including maintaining upper airway patency during inspiration (24–26). Damage to the phrenic and XII nerves can lead to respiratory insufficiency, dysphagia, aspiration pneumonia, and eventually respiratory failure (2, 14, 18). Despite the significant respiratory pathology, the mechanism by which polyQ-ataxin-7 causes this respiratory pathology remains unknown. The primary aim of this study was to investigate the impact of polyQ-ataxin-7 protein accumulation in the glia and motor neurons of the XII and putative phrenic motor nuclei. The secondary aim was to investigate the impact of this pathology on efferent nerve output and neuromuscular junction (NMJ) integrity. Using the SCA7 mouse model, we report changes in gene expression in the medulla and cervical spinal cord, histopathology in the XII and putative phrenic motor nuclei, the XII and phrenic nerves, and the NMJ.

## Results

### Expression of ataxin-7 is prominent in the astrocytes of the XII and phrenic respiratory motor nuclei and results in increased cell death.

**Figure 1:**
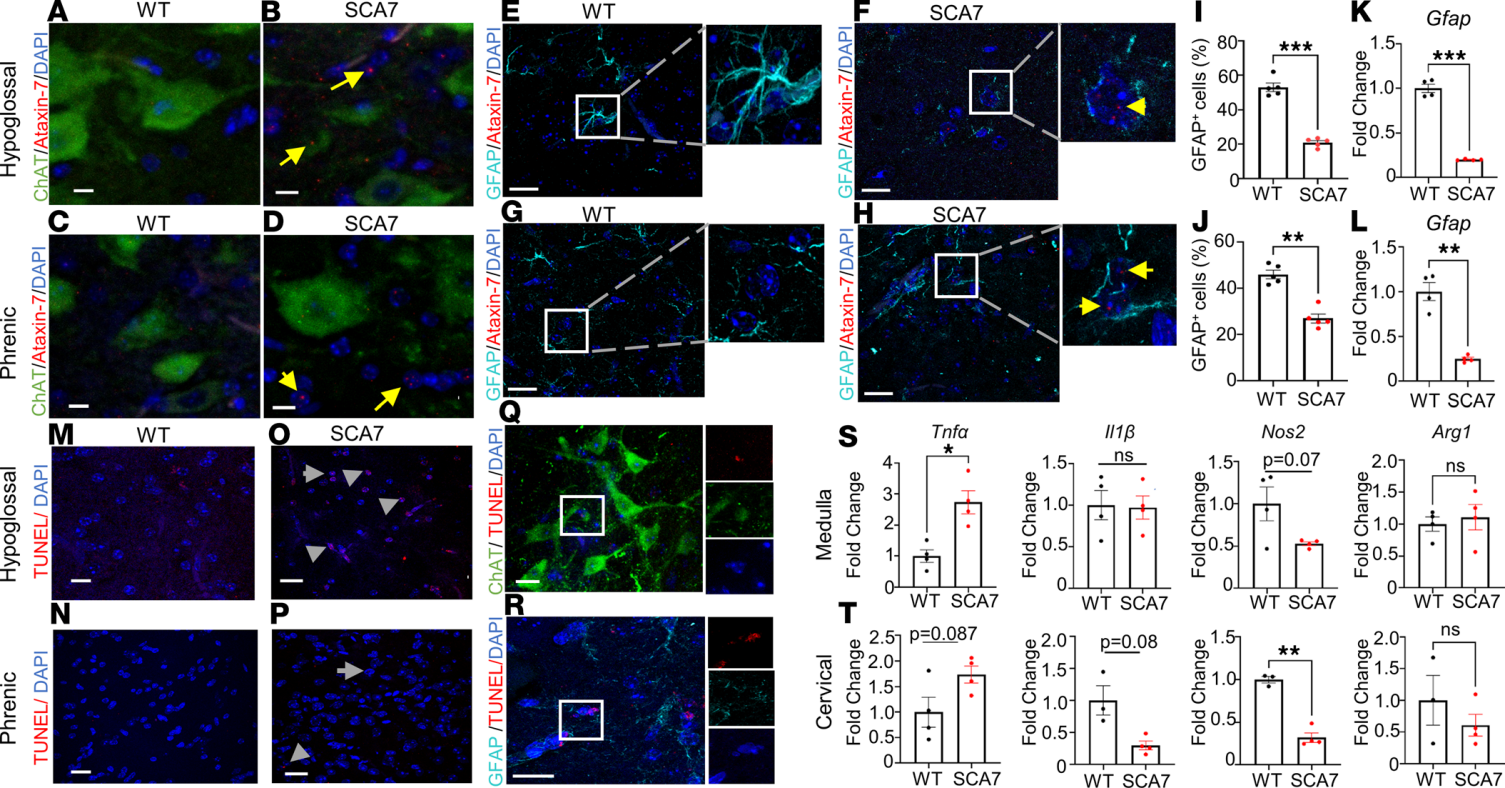
Accumulation of mutant ataxin-7 in the XII and phrenic respiratory control centers is associated with increased cell death and reduced proinflammatory cytokine expression. (**A**–**H**) Representative confocal images from 9-week WT (*n* = 4) and SCA7 XII (*n* = 4) (**A** and **B**, and **E** and **F**) and putative phrenic (**C** and **D**, and **G** and **H**) respiratory centers immunostaining for anti-ChAT (green)/anti–ataxin-7 (red) (**A**–**D**), and anti-GFAP (cyan)/anti–ataxin-7 (red) (**E**–**H**). Cell nuclei were visualized by DAPI (blue). The yellow arrow indicates an accumulation of ataxin-7. Scale bars: 10 μm. (**I** and **J**) Quantification of GFAP^+^DAPI^+^ cells (in percentage) in XII (**I**) and putative phrenic (**J**) respiratory centers from WT (*n* = 4) and SCA7 (*n* = 4) mice. **P < 0.001, ***P < 0.001 by 2-tailed Student’s *t* test. (**K** and **L**) Expression of *Gfap* in the medulla (**K**) and cervical spinal cord (**L**) from WT (*n* = 4) and SCA7 (*n* = 4) mice by qPCR. **P < 0.001, ***P < 0.001 by unpaired, 2-tailed Student’s *t* test. (**M**–**P**) Representative confocal images from 9-week WT (*n* = 4) (**M** and **N**) and SCA7 (*n* = 4) (**O** and **P**) XII (**M** and **O**) and phrenic (**N** and **P**) respiratory control centers stained with TUNEL (red). Cell nuclei were visualized by DAPI (blue). Scale bars: 20 μm. (**Q** and **R**) Representative confocal images from 9-week SCA7 (*n* = 2) XII respiratory center immunostained for anti-ChAT (green)/TUNEL (red) (**Q**) and anti-GFAP (cyan)/TUNEL (red) (**R**). Cell nuclei were visualized by DAPI (blue). Scale bars: 20 μm. (**S** and **T**) Expression of proinflammatory (*Tnfa*, *Il1b*, *Nos2*) and antiinflammatory markers (*Arg1*) in the medulla (**S**) and cervical spinal cord (**T**) from WT (*n* = 4) and SCA7 (*n* = 4) mice. All data presented as mean ± SEM. *P < 0.05, **P < 0.001 by 2-tailed Student’s *t* test.

Similar to infantile-SCA7 patients, the infantile mouse model of SCA7 demonstrates respiratory insufficiency associated with episodes of erratic breathing and prolonged apneic events at the young age of 8 weeks (21). PolyQ expansion of ataxin-7 is known to promote the accumulation of the ataxin-7 protein (20, 27). Infantile-onset SCA7 mice exhibit intranuclear as well as cytoplasmic accumulation of ataxin-7 in the XII and phrenic respiratory control centers (21). Since motor neurons (28) and astrocytes (29, 30) both play a critical role in regulating respiratory physiology, we sought to determine whether pathological polyQ-ataxin-7 accumulates in these cells within the XII and phrenic respiratory motor nuclei. We performed coimmunostaining of the medulla and cervical spinal cord from 9-week WT and SCA7 (at the preterminal) mice with anti–ataxin-7 and either anti-GFAP (for astrocytes) or anti-ChAT (for motor neurons) antibodies. Ataxin-7 intranuclear accumulation was robust in the cells surrounding the XII and putative phrenic motor neurons, with some evidence of cytoplasmic ataxin-7 in the XII motor neurons (Figure 1, A–D). We found extensive intranuclear inclusions of ataxin-7 in the astrocytes surrounding both XII (Figure 1, E and F) and putative phrenic respiratory centers (Figure 1, G and H). In addition, when quantified for astrocytes (GFAP^+^ cells), we found that the number of astrocytes was significantly reduced in XII (*P* = 0.0003) (Figure 1I) and phrenic motor control centers (*P* = 0.0024) (Figure 1J). Similarly, the expression of *Gfap* transcripts quantified by quantitative PCR (qPCR) was significantly decreased in both the medulla (*P* = 0.0011) and cervical spinal cord (*P* = 0.0059) (Figure 1, K and L).

To evaluate the impact of ataxin-7 expression on neurodegeneration, we performed TUNEL staining and confirmed extensive cell death in the XII motor neuron pool (Figure 1, M and O) and putative phrenic motor neurons (Figure 1, N and P). However, cell death was more extensive in the medulla than in the cervical spinal cord. We found that the TUNEL-positive cells colocalized with both ChAT^+^ and GFAP^+^ cells at the XII motor neuron pool in the medulla of SCA7 mice (Figure 1, Q and R). This suggests that the presence of pathological polyQ-ataxin-7 in SCA7 mice can result in the loss of both motor neurons and astrocytes. Since inflammatory pathways can influence cell death, we probed for proinflammatory and antiinflammatory cytokines (31). Proinflammatory cytokine–like TNF-α plays a critical role in cell death and neuroinflammation that results in the pathogenesis of several neurodegenerative diseases, including multiple sclerosis, Parkinson disease, and Alzheimer disease (32–35). Interestingly, we found a significant increase in the expression of *Tnfa* in the medulla (*P* = 0.038) (Figure 1S), but a modest increase in the cervical spinal cord (*P* = 0.121) by qPCR (Figure 1T). In contrast, there were significant reductions in proinflammatory cytokine transcripts such as *Il1b* (*P* = 0.08) and *Nos2* (*P* = 0.004) in the cervical spinal cord (Figure 1T), but no differences in their expression in the medulla *Il1b* (*P* = 0.817) and *Nos2* (*P* = 0.074) (Figure 1S). No difference in the expression of the antiinflammatory cytokine *Arg1* was observed in the medulla (*P* = 0.38) and cervical spinal cord (*P* = 0.6).

### Expression of pathological ataxin-7 in SCA7 mice results in altered transcription of genes in the medulla and cervical spinal cord by 9 weeks of age.

**Figure 2:**
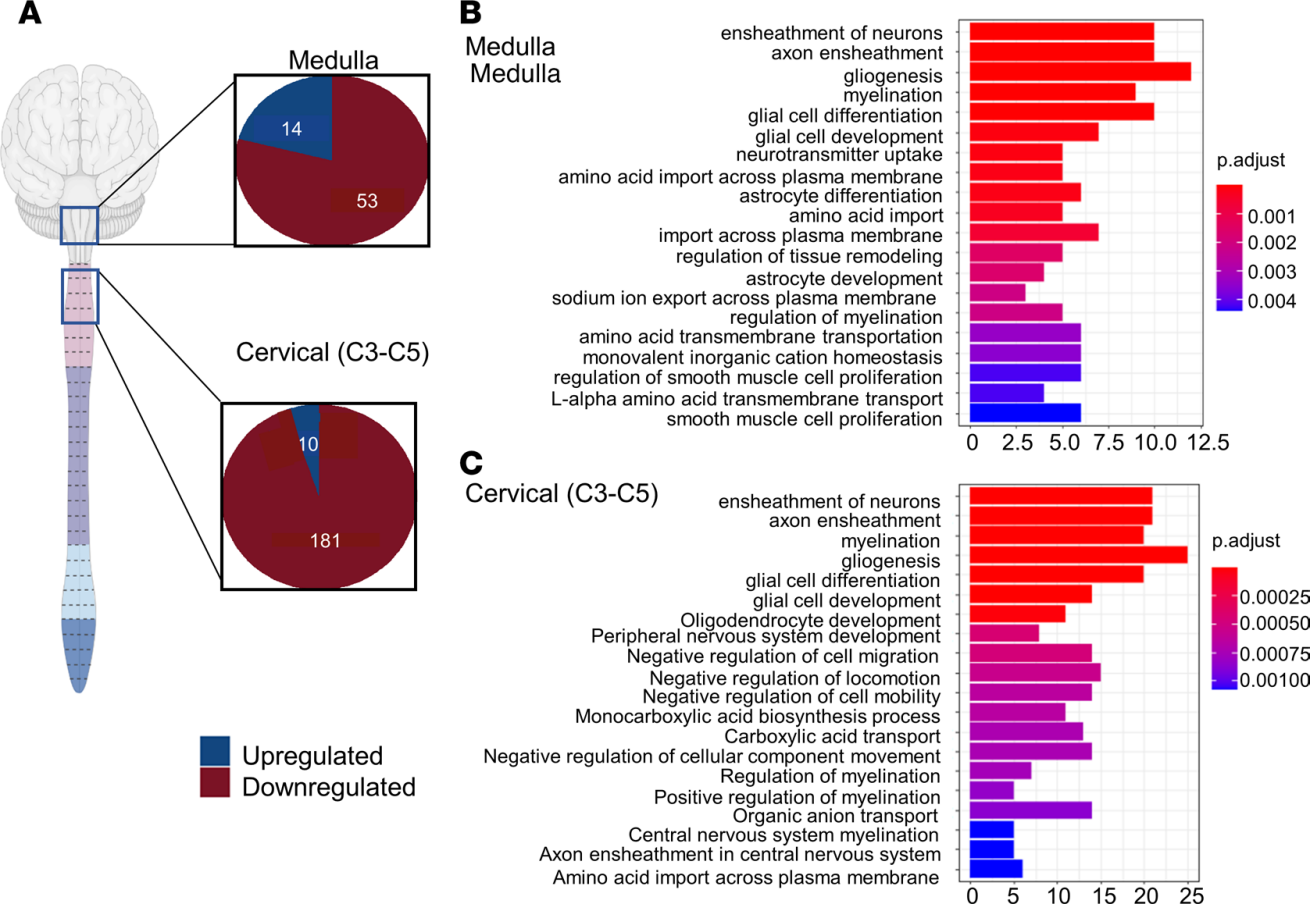
Bulk RNA seq analysis of medulla and cervical of SCA7 exhibits differentially expressed genes that alter biological pathways. (**A**) Bulk RNA analysis of the medulla and cervical spinal cord (*n* = 5 WT and 5 SCA7) shows upregulated and downregulated genes in 9-week SCA7 mice compared with WT mice. (**B** and **C**) GO enrichment analysis of 20 most differentially regulated biological pathways in the medulla (**B**) and cervical spinal cord (**C**) of 9-week WT and SCA7 (*n* = 5 WT and 5 SCA7) mice. The *x* axis marks the enrichment score with a significance cutoff point of *P* = 0.01.

Ataxin-7 is a transcription factor that is a core component of the STAGA coactivator complex, which regulates cell signaling and plays a critical role in transcription (7, 36). To understand the implication of the intranuclear expression of mutant ataxin-7, we investigated the gene expression profile of the medulla and C3–C5 of the cervical spinal cord at P8 (neonates), 5 weeks (presymptomatic), and the end stage of the mice — 9 weeks (preterminal) — in SCA7 and WT mice. Relative to 9-week WT control littermates, RNA-seq data from the 9-week SCA7 mice revealed 67 and 191 differentially expressed genes in the medulla and cervical spinal cord, respectively. Specifically, in the medulla, 14 genes were upregulated and 53 genes were downregulated, while in the cervical spinal cord, 10 genes were upregulated and 181 genes were downregulated (Figure 2A). Differentially expressed genes involved in each enriched pathway are described in Supplemental Tables 1 and 2 (supplemental material available online with this article; https://doi.org/10.1172/jci.insight.170444DS1).

To gain insight into SCA7 pathology, we clustered differentially expressed genes between 9-week WT and SCA7 mice. Enrichment analysis was executed based on functional similarity and visualized for the top 20 differentially regulated biological pathways in the medulla (Figure 2B) and cervical spinal cord (Figure 2C) using Gene Ontology (GO) enrichment analysis (*n* = 5 per genotype). In both tissues, the top 5 altered pathways in SCA7 mice were similar and included pathways that regulate the ensheathment of neurons and axons, myelination, gliogenesis, glial cell development, and glial cell differentiation.

### Reduced expression of glutamate and glycine transporters in the medulla and cervical spinal cord of SCA7 mice.

**Figure 3:**
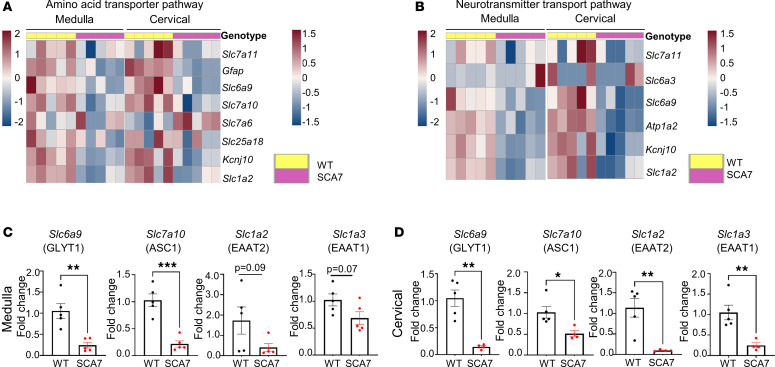
Accumulation of mutant ataxin-7 alters amino acid transport and neurotransmitter activity in the medulla and cervical spinal cord. (**A** and **B**) Heatmaps showing differentially expressed genes regulating amino acid transporter (**A**) and neurotransmitter (**B**) activity pathways in the medulla and cervical spinal cords of 9-week WT (*n* = 5) and SCA7 (*n* = 5) mice. (**C** and **D**) Expression of *Slc6a9*, *Slc7a10*, *Slc1a2*, and *Slc1a3* in the medulla (**C**) and cervical spinal cord (**D**) in 9-week WT (*n* = 5) and SCA7 (*n* = 5) mice analyzed by qPCR. Data presented as mean ± SEM. *P < 0.05, **P < 0.001, ***P < 0.001 by 2-tailed Student’s *t* test.

RNA-seq data from 9-week WT and SCA7 mice show differential expression of amino acid transporters involved in amino acid transportation (Figure 3A) and neurotransmitter uptake activity (Figure 3B) in the medulla and cervical spinal cord. We found that expression of *Slc1a2* (EAAT2/GLT1), a glutamate transporter, was significantly diminished in the SCA7 medulla (*P* = 0.09) and cervical spinal cord (*P* = 0.005), which was validated by qPCR (Figure 3, C and D). In addition, *Slc1a3* (EAAT1/GLAST) — another glutamate transporter — was also reduced in the SCA7 mouse medulla (*P* = 0.072) and cervical spinal cord (*P* = 0.0191) (Figure 3, C and D). This reduction is consistent with previously reported findings in the cerebellum of the SCA7 mouse model (37). Reduction in glutamate transporters results in an excess buildup of glutamate in the synaptic cleft that results in neuronal death (38, 39). Therefore, we speculate that the lack of glutamate transporters *Slc1a2* and *Slc1a3* leads to significant cell death, as shown in Figure 1, M–P. Glycine transporters such as glycine transporter type-1 (GlyT1), encoded by *Slc6a9*, and alanine-serine-cysteine-1 transporter (ASC-1), encoded by *Slc7a10*, impact the amplitude and frequency of respiratory neuronal output. GlyT1 regulates the rate of breathing (40), while ASC-1 modulates presynaptic glycine levels that impact respiratory rhythm (41, 42). Interestingly, RNA-seq analysis revealed decreased expression of both *Slc6a9* and *Slc7a10* genes in the medulla and cervical spinal cord of the SCA7 mice relative to the WT mice (Figure 3, A and B). When verified by qPCR, expression of both *Slc6a9* and *Slc7a10* was reduced in the medulla (*P* = 0.0025, *P* = 0.0003) and cervical spinal cord (*P* = 0.0012, *P* = 0.015) at 9 weeks of age. No significant differences in these genes were observed in pups and 5-week mice (Supplemental Figure 2, A and B). These *P* values correspond to the data generated by qPCR, while the *P* values of differentially expressed genes derived from heatplot-generated RNA-seq data are in Table 1 and Table 2.

### SCA7 mice exhibit XII and phrenic motor axonopathies.

**Figure 4:**
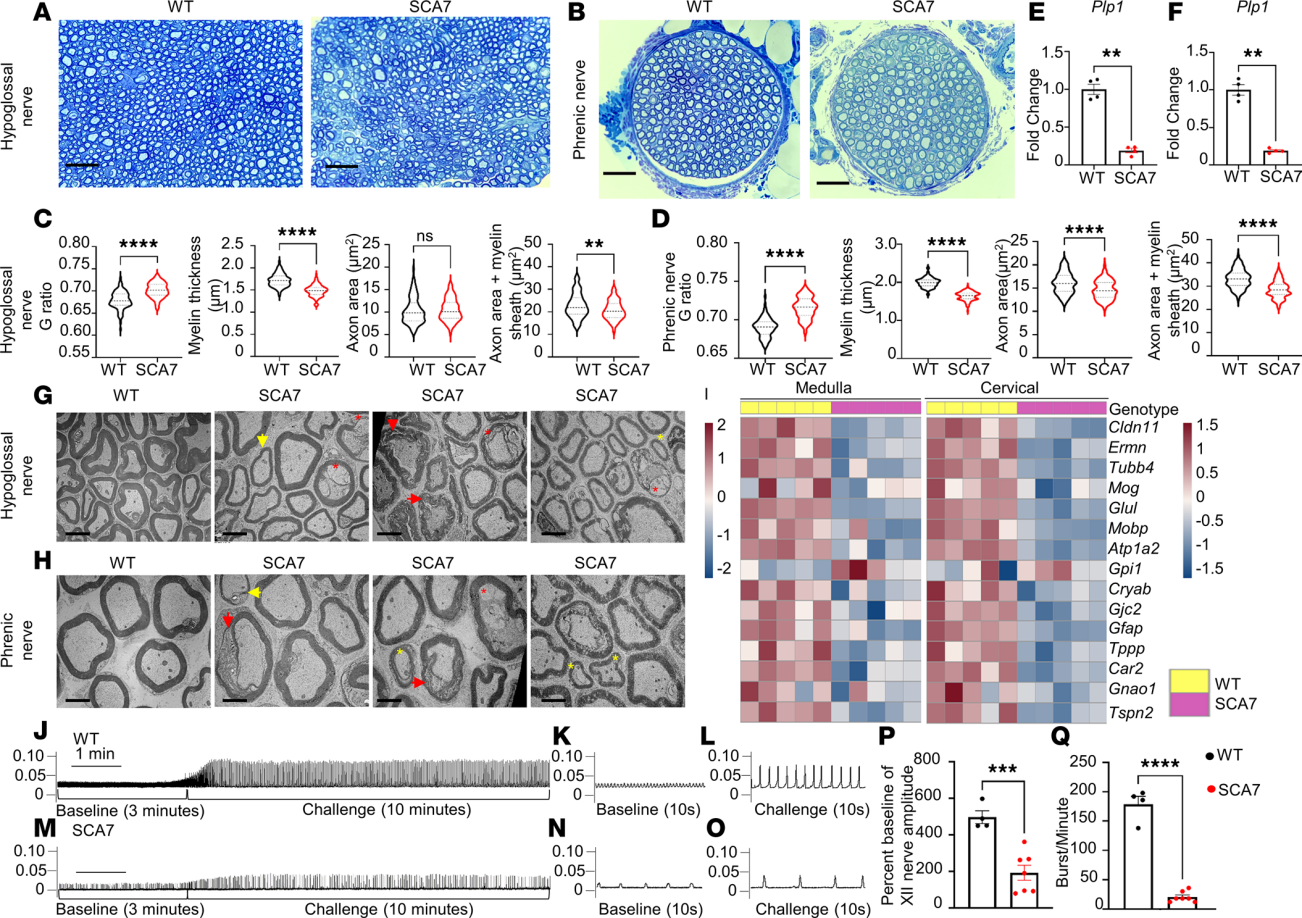
SCA7 mice exhibit significant XII and phrenic nerve pathology. (**A** and **B**) Representative bright-field images of 9-week WT (*n* = 4) and SCA7 (*n* = 4) toluidine blue–stained XII (**A**) and phrenic (**B**) nerves. Scale bars: 30 μm. (**C** and **D**) Graphical representation of g-ratio, myelin thickness, axon area, and total axon area with myelin sheath of XII nerve (**C**) in 9-week WT (*n* = 5) and SCA7 (*n* = 7) mice and phrenic nerve (**D**) in 9-week WT (*n* = 6) and SCA7 (*n* = 7) mice. (**E** and **F**) Expression of *Plp1* by qPCR in the medulla (**E**) and cervical spinal cord (**F**) of 9-week WT (*n* = 4) and SCA7 (*n* = 4) mice. (**G** and **H**) Transmission electron microscopy images of XII (**G**) and phrenic (**H**) nerves from 9-week WT (*n* = 4) and SCA7 (*n* = 4) mice. Yellow arrows indicate demyelination, red arrows indicate decompaction of the myelin sheath, yellow asterisks indicate smaller sized axons, and red asterisks indicate accumulation of vacuoles in the axons and Schwann cells surrounding the axons. Scale bars: 2 μm. (**I**) Heatmap highlights differentially expressed genes regulating myelin sheath activity pathway in 9-week WT (*n* = 5) and SCA7 (*n* = 5) medulla and cervical spinal cord (C3–C5 region of the spinal cord). (**J**–**O**) Representative traces of WT (**J**–**L**) and SCA7 (**M**–**O**) XII nerve recordings at baseline (50% O_2_, 50% N_2_) and during a respiratory challenge (7% CO_2_, 21% O_2_/N_2_ balance). Expanded time-scale traces of the 10-second recording are shown for baseline (**K** and **N**) and challenge (**L** and **O**). (**P**) Graphical representation of XII nerve amplitude at baseline. The graph represents percentage of baseline for the amplitude of XII nerve amplitude of WT (*n* = 4) and SCA7 (*n* = 5) mice. Percentage of baseline was calculated as (challenge – baseline)/baseline × 100. (**Q**) Graphical representation of the frequency of XII nerve burst at baseline of WT (*n* = 4)and SCA7 (*n* = 5) mice. Data presented as mean ± SEM. **P < 0.01, ***P < 0.001, ****P < 0.0001 by 2-tailed Student’s *t* test.

Next, we examined the XII and phrenic nerves in 9-week WT and SCA7 mice (*n* = 4/genotype) to determine whether the transcriptional changes due to polyQ-ataxin-7 expression result in nerve pathology. Toluidine blue staining of the XII (Figure 4A) and phrenic (Figure 4B) nerves demonstrated variability in axon size and myelin staining intensity (in blue) in SCA7 mice compared with WT mice. These images were used to quantify the g-ratio (diameter of axon/diameter of axon + myelin), myelin thickness, and axon area of XII (Figure 4C) and phrenic (Figure 4D) nerves. The g-ratio of both these nerves was significantly increased, with diminished myelin thickness of these nerves in 9-week SCA7 mice relative to age-matched and sex-matched WT mice. The axonal area of the phrenic nerves was also decreased in SCA7 mice, while the XII axonal area remained intact. However, the total axon size with myelin sheath was significantly decreased in both XII and phrenic nerves. Electron microscopic images of the XII (Figure 4G) and phrenic nerves (Figure 4H) further confirmed the extent of the pathology. Both XII and phrenic nerves from SCA7 mice demonstrated severe demyelination (indicated by a yellow arrow), decompaction of the myelin sheath (indicated by a red arrow), smaller axons (yellow asterisk), and abnormal vacuole formation in the axons as well as Schwann cells encompassing myelin sheath of axons (indicated by red asterisk).

To investigate the mechanism involved in demyelination, we utilized RNA-seq data to examine the differentially expressed genes involved in myelination activity in the medulla and cervical spinal cord of 9-week (Figure 4I), 5-week, and neonatal (P8) (Supplemental Figure 2C) WT and SCA7 mice. The *P* values of differentially expressed genes from the RNA-seq data are provided in Table 3. As shown in the heatmap in Figure 4I, the genes essential for optimal myelination were reduced in the medulla and cervical spinal of SCA7 mice. However, there was no difference in the expression of genes involved in myelination between P8 and 5-week WT and SCA7 mice (Supplemental Figure 2C). Furthermore, we found that the expression of proteolipid protein 1 (*Plp1*) was significantly reduced in SCA7 mice in both the medulla (*P* = 0.0005) and cervical spinal cord (*P* = 0.0023) by qPCR (Figure 4, E and F). Based on RNA-seq data, the expression of the *Plp1* gene exhibited a log_2_(fold change) of approximately 1.7 in the medulla (*P* = 1.61 × 10^–5^) and approximately 2.6 in the cervical spinal cord (*P* = 2.94 × 10^–18^). Plp1 is the most abundant membrane protein in CNS myelin (43), and *Plp1* mutations are linked to multiple neurological disorders (44, 45). Genetic loss of *Plp1* in oligodendrocytes causes axonopathy and secondary neuroinflammation (46). Altogether, these data confirm consistent demyelination in SCA7 XII and phrenic nerves in the preterminal stage of the disease.

To determine whether these transcriptional and histological changes affected efferent nerve output, we studied the XII nerve efferent output at eupnea and during a hypercapnic challenge (Figure 4, J–O). Representative XII nerve tracings are shown for WT (Figure 4, J–L) and SCA7 (Figure 4, M–O) mice at baseline as well as during the hypercapnic challenge. WT mice recorded a consistent response during baseline (Figure 4K) and could mount a significant response to the hypercapnic challenge (Figure 4L). In contrast, the SCA7 mice exhibited significantly blunted nerve efferent output at baseline (Figure 4N) and during the hypercapnic challenge (Figure 4O). Inspiratory burst amplitude was considerably lower in SCA7 mice than in WT mice (*P* = 0.008) (Figure 4P). The frequency of XII nerve bursts was also significantly attenuated in the SCA7 mice at baseline relative to WT mice (*P* = 0.012) (Figure 4Q). These data suggest that SCA7 mice have XII nerve pathologies that reduce nerve function at baseline and during hypercapnia.

### SCA7 mice exhibit NMJ pathology, with increased neurofilament expression.

**Figure 5:**
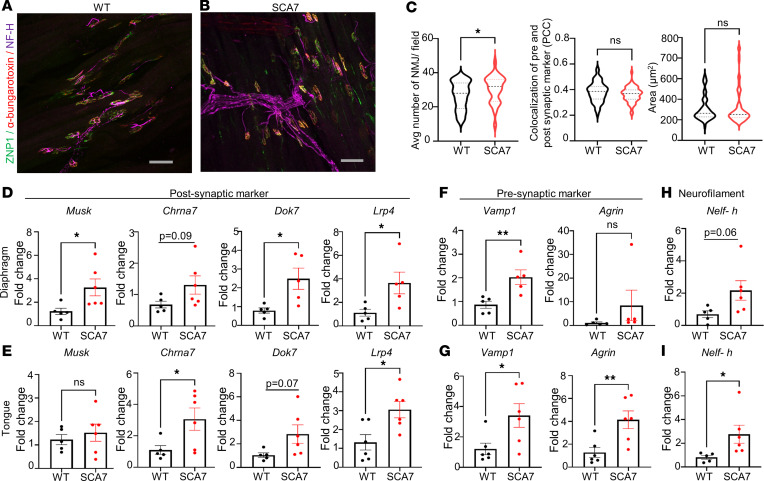
Increase in neuromuscular junction (NMJ) clusters and higher expression of synaptic markers in SCA7 mice. (**A** and **B**) Representative confocal images of 9-week WT (**A**) and SCA7 (**B**) diaphragms labeled with anti-ZNP1 (green, presynaptic marker), α-bungarotoxin (red, postsynaptic marker), and anti–neurofilament heavy chain (anti–NF-H, purple). Scale bars: 10 μm. (**C**) Graphical representation of the average number of NMJ clusters collected from 5 different fields from WT (*n* = 6) and SCA7 (*n* = 6) mice, colocalization of presynaptic and postsynaptic markers using Pearson’s correlation coefficient in WT (*n* = 5) and SCA7 (*n* = 5) mice, and area of endplates of WT (*n* = 6) and SCA7 (*n* = 6) mice at 9 weeks of age. (**D** and **E**) Expression of postsynaptic makers (**F** and **G**), presynaptic markers (**H** and **I**), and neurofilament in the diaphragm and tongue of 9-week WT (*n* = 5) and SCA7 (*n* = 6) mice. Data presented as mean ± SEM. *P < 0.05, **P < 0.01 by 2-tailed Student’s *t* test.

The NMJ is a synaptic connection between the terminal end of a motor nerve (presynaptic) and a muscle fiber (postsynaptic). To assess whether SCA7 mice exhibit NMJ pathology in respiratory muscles, we examined the NMJ clusters (presynaptic and postsynaptic components) in the diaphragm and tongue of WT and SCA7 mice. Immunohistochemical analysis of 9-week WT and SCA7 mouse diaphragms is shown in Figure 5, A and B, and did not reveal any differences between colocalization of presynaptic and postsynaptic membranes. However, we observed a significant increase in the number of NMJ clusters in SCA7 mice (Figure 5C). Next, we evaluated the expression of postsynaptic (*Musk*, *Lrp4*, *Dok7*, *Chrna7*) and presynaptic marker (*Vamp1*, *Agrin*) mRNA in both the tongue and diaphragm of 9-week (preterminal) and 5-week (presymptomatic) WT and SCA7 mice by qPCR. At 9 weeks of age, SCA7 mice exhibited a significant increase in the expression of the presynaptic and postsynaptic markers in the diaphragm (Figure 5, D and F) and tongue (Figure 5, E and G). We observed a similar trend in the expression of these markers in the diaphragm (Supplemental Figure 3, A and C) and tongue (Supplemental Figure 3, B and D) of 5-week SCA7 mice relative to WT mice. The neurofilament heavy chain was aggregated and swollen in SCA7 (Figure 5B) diaphragms in contrast to those of WT mice (Figure 5A). These data are consistent with the increase in transcripts of neurofilament heavy chain in the diaphragms and the tongues of SCA7 mice (Figure 5, H and I). In summary, these data imply that transcriptional modifications occur in the respiration-associated muscles before the onset of symptoms. The increase in the number of NMJ clusters and overexpression of synaptic markers in SCA7 mice suggests a compensatory mechanism induced by the muscles and axon terminals to offset the decreased nerve impulse caused by CNS-specific pathology.

**Figure 6:**
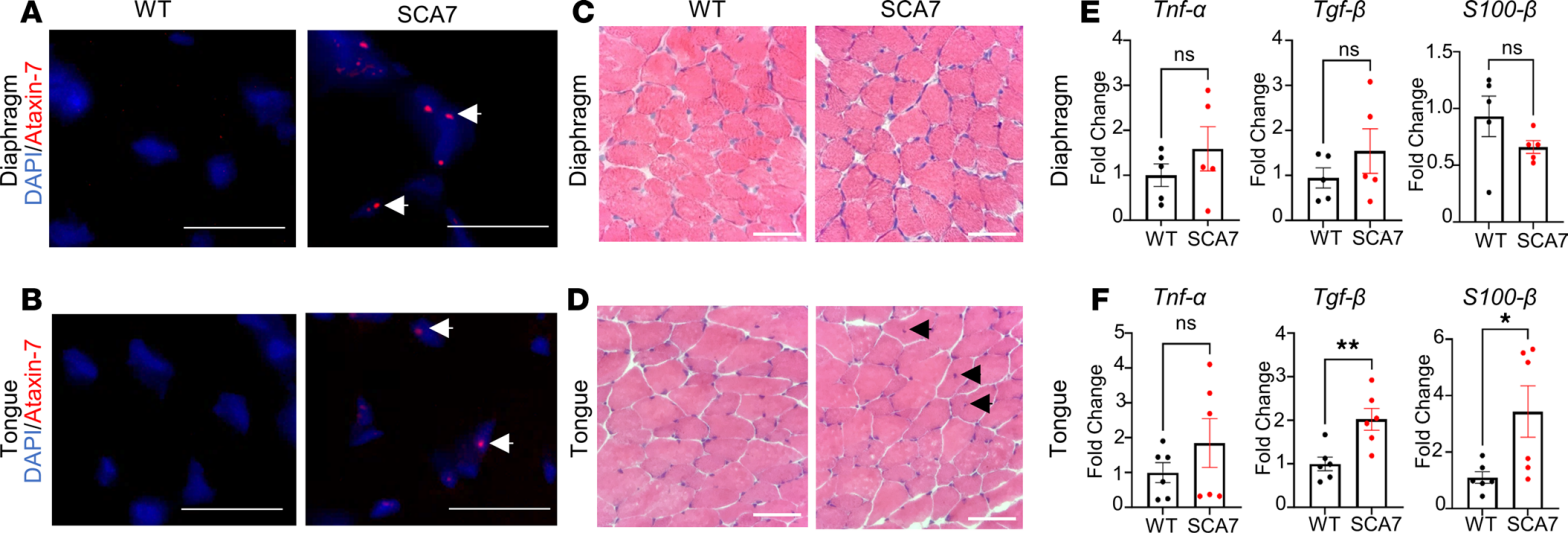
Intranuclear accumulation of ataxin-7 in diaphragm and tongue. (**A** and **B**) Representative images of 9-week WT and SCA7 diaphragm (**A**) and tongue (**B**) labeled with anti–ataxin-7 (red) and DAPI (blue). Scale bars: 90 μm. (**C** and **D**) Representative images of H&E-stained diaphragm (**C**) and tongue (**D**) of 9-week WT and SCA7 (*n* = 4/genotype). Black arrows show centralization of nuclei. Scale bars: 45 μm. (**E**) Expression of *Tnfa*, *Tgfb*, and *S100b* transcripts in 9-week WT and SCA7 diaphragm (*n* = 5/genotype) (**E**) and tongue (*n* = 6/genotype) (**F**).

To further determine whether ataxin-7 accumulates in muscles essential for breathing and contributes toward myopathogeneis, we stained the diaphragm and genioglossus muscles of the tongue from WT and SCA7 mice for ataxin-7 (Figure 6, A and B). We could not detect ataxin-7 in WT muscle tissues; however, in SCA7 muscle tissues ataxin-7 was observed in the nuclei of muscle cells. Centralization of nuclei in muscle fibers is a marker of regeneration and is associated with myopathy (47, 48), which can be detected by hematoxylin and eosin (H&E) staining. H&E staining of muscles revealed no differences between WT and SCA7 diaphragm (Figure 6C). In contrast, we observed the centralization of nuclei in the SCA7 genioglossus muscles of the tongue, without any infiltration of immune cells (Figure 6D). We further examined whether the muscles undergo inflammation, fibrosis, and repair mechanisms in WT and SCA7 diaphragm (Figure 6E) and tongue (Figure 6F). We evaluated the mRNA level of *Tnfa*, *Tgfb*, and *S100b* in the muscles. Expression of *Tnfa* was not different in the diaphragm (*P* = 0.84) as well as in the tongue (*P* = 0.39) between WT and SCA7 mice (Figure 6, E and F). Also, the expression of *Tgfb* mRNA was similar in the diaphragm of WT and SCA7 mice (*P* = 0.54) (Figure 6, E and F). *Tgfb* elevation is a marker for fibrosis as well as required for myogenic differentiation and muscle repair (49–51). However, it was significantly higher in SCA7 tongue than that of WT (*P* = 0.009) (Figure 6F), which indicates muscle regeneration or elevated fibrosis in tongue (50, 51). In addition, when we probed for S100β (*S100b*), member of the S100 family of Ca^2+^-binding proteins, we found that the expression was much elevated in the tongue of SCA7 mice than compared with WT mice. No differences were recorded in WT and SCA7 diaphragm.

## Discussion

This study is the first to our knowledge to highlight the significant histological and cellular disruptions that occur as a result of the expression of polyQ-ataxin-7 in the medulla and cervical spinal cord of an infantile model of SCA7 mice. Specifically, SCA7 results in altered expression of multiple transcripts in the medulla and cervical spinal cord, contributes to the inactivation of glial cells, and disrupts multiple signaling pathways required for myelination, amino acid transport, and neurotransmitter uptake. Ultimately, expression of pathological polyQ-ataxin-7 leads to neurodegeneration of XII and phrenic motor neurons, demyelination, and diminished activity of the XII and phrenic nerves. Furthermore, glycine transporters, essential for breathing, and glutamate transporters, required for synaptic health maintenance, are significantly reduced in the medulla and cervical spinal cord of SCA7 mice.

### Localization of ataxin-7 in the medulla and cervical spinal cord.

The subcellular localization and abundance of normal and mutant ataxin-7 are controlled in a regionally specific way (52, 53). The distribution of ataxin-7 changes dynamically due to its capability of shuttling between nuclei and cytoplasm (8, 54, 55). In the cerebellum, non–cell-autonomous neurodegeneration results in SCA7 pathology (37, 56, 57). Specifically, pathological ataxin-7 aggregates in the Bergman glia, Purkinje cells, and inferior olive in the cerebellum and disrupts the interdependent neural-glial signaling essential for the survival and function of all cells. In the medulla, neurons of SCA7 patients exhibit cytoplasmic ataxin-7, and show intranuclear ataxin-7 accumulation and neurodegeneration (58), whereas in healthy patients ataxin-7 is expressed within the cytoplasm of neurons and does not excessively accumulate in the nucleus. Specifically, in the XII motor neurons, SCA7 patients exhibit neurodegeneration and neuronal intranuclear ataxin-7 accumulation (18). In our SCA7 mouse model, we also found expression of ataxin-7 in the XII motor neurons as well as in the putative phrenic motor neurons. However, the ataxin-7 accumulation was more prevalent in the cytoplasm of these neurons. In contrast, the astrocytes in these motor pools demonstrated intranuclear ataxin-7 expression. Ataxin-7 is more abundant in astrocytes in the CNS compared with other glia and neurons in humans and mice (59, 60). Astrocytes play an important role in the respiratory control centers and are essential for the regulation of breathing (30).

### Altered transcription in SCA7 causes neurodegeneration and blunted XII nerve response.

Compared with WT mice, SCA7 mice had significant demyelination and decompaction of myelin in both the XII and phrenic nerves (Figure 4). The expression of pathological ataxin-7 results in the downregulation of genes important for myelination, including *Plp1*, *Mog*, and *Mobp*. In addition, there was a significant decrease in the efferent nerve output of the XII nerves in the SCA7 mice compared with WT controls. This diminished nerve output is likely due to a combination of demyelination and XII motor neuron degeneration. Several neurological disorders are associated with demyelination (61), causing neuronal damage and adversely affecting synapsis.

Furthermore, the expression of *Slc6a9* (GlyT1), which regulates glycine levels, was diminished in the medulla and cervical spinal cord of the SCA7 mice. *Slc6a9* plays an essential role in breathing. This gene is involved in both amino acid transport and neurotransmitter uptake activity. *Slc6a9*-deficient mice typically demonstrate a short life span (6–14 hours after birth) and depressed breathing pattern with attenuated frequencies of breath (40), similar to what is seen in the SCA7 mouse model. This highlights the significance of this glycine transporter in respiratory control. Meanwhile, *Slc7a10* (ASC-1), which was also significantly reduced in SCA7 mice, is considerably enriched in the brain and spinal cord. It plays a critical role in modulating presynaptic glycine levels that can regulate the respiratory rhythm generation in the brain (41). *Slc7a10*-null mice have lower glycine levels in the spinal cord and diminished amplitude of glycinergic postsynaptic currents in motor neurons (42). Our findings thus point to a potential mechanism for respiratory dysfunction in SCA7; downregulation of *Slc6a9* and *Slc7a10* disrupts glycinergic signaling and diminishes the amplitude of glycinergic postsynaptic currents in motor neurons (42), which in turn results in decreased respiratory motor output.

The loss of astrocytes in SCA7 mice reduces the expression of astrocyte-specific gene expression. In addition, as previously shown, the polyQ expansion decreases the *ATXN7* occupancy and can modulate gene expression and chromatin modification in human astrocytes, causing non–cell-autonomous neurodegeneration in SCA7 (62–64). Therefore, the loss of astrocytes as well as intranuclear accumulation of pathological polyQ-ataxin-7 that results in transcriptional dysregulation in the astrocytes can affect the expression of astrocyte-specific genes. In this study, we also noted a decrease in the astrocyte-specific glutamate transporters EAAT1 and EAAT2 that regulate neuronal health and synapsis (65, 66). Attenuated expression of glutamate transporters causes accumulation of glutamate in the synaptic cleft, which leads to neuronal death (38, 39). These findings can explain the loss of XII and phrenic motor neurons in SCA7 mice in the medulla and cervical spinal cord, respectively (21). In addition, the glycine transporter GlyT1 (*Slc6a9*), which is mostly expressed by astrocytes (67, 68), was significantly reduced in both the SCA7 medulla and cervical spinal cord. Lack of GlyT1 results in prolonged periods of apnea with an overall decreased burst frequency in mice and ultimately leads to an early death by respiratory failure. (40). Thus, based on our data, we speculate that intranuclear accumulation of pathological polyQ-ataxin-7 in astrocytes can lead to death and therefore results in altered expression of multiple genes responsible for the regulation of breathing.

The pre-Bötzinger complex (pre-BötC) is a neural network located in the medulla, essential for the generation and modulation of respiratory rhythm (69). The excitation of pre-BötC neurons also influences XII motoneuron firing to maintain upper airway patency during breathing (70). The central respiratory chemoreceptor (CRC) cells are sensitive to changes in CNS PCO2 or pH and contribute to changes in the frequency of breathing during hypercapnia (71). Activation of the pre-BötC and CRC cells in the medulla is regulated by astrocytes and plays an important role during hypercapnic and hypoxic conditions (30, 72–74). In this study, we observed the loss of astrocytes and astrocyte-specific gene expression in XII motor neuron pools in the medulla. There is a possibility that the loss of astrocytes and/or intranuclear accumulation of ataxin-7 in astrocytes at pre-BötCs or in CRC cells can lead to either dysregulated respiratory center rhythm generation or chemosensation under hypercapnic conditions. This may explain the marked difference in the frequency of XII nerve bursting in hypercapnic conditions in SCA7 mice and needs to be further studied.

### The NMJ is altered in the respiratory muscles of SCA7.

NMJs are specialized synapses in the peripheral nervous system that permit communication between the motor nerve terminal and skeletal muscle fibers. We found that colocalization between the pre- and postsynaptic membranes did not significantly differ between WT and SCA7 mice. However, there was an increased expression of neurofilaments in SCA7 mice. Neurofilaments determine the axonal caliber, control signal conduction, regulate the transport of synaptic vesicles, and modulate synaptic plasticity by binding to neurotransmitter receptors (75). An upregulation of neurofilament production occurs in multiple neurological disorders (76–78). Specifically, increased accumulation of the neurofilament is associated with slow and delayed NMJ maturation and maintenance (79). In addition, increased concentration of neurofilament in the cerebrospinal fluid and plasma occurs in SCA7 patients, as well as in several neurodegenerative disorders such as ALS, Huntington disease, Alzheimer disease, and other related SCA disorders (SCA1, SCA2, SCA3) (80).

The clustering of acetylcholine receptors on the postsynaptic muscle membrane is required for optimal synapse formation and maintenance of the NMJ. This clustering is regulated by agrin and the postsynaptic receptor complex, which consists of muscle-specific kinase (MuSK), low-density lipoprotein receptor–related protein 4 (LRP4), and docking protein 7 (DOK7) (81). Interestingly, we observed an increased level of *Agrin*, *Musk*, *Lrp4*, and *Dok7* in SCA7 mice. We speculate that this overexpression of postsynaptic transcripts is an attempt to maintain homeostasis through overcompensation in the muscle for pathology in the neurons and nerves. Retrograde signaling from muscles is essential for synapse development and the release of neurotransmitters (82). The muscle signals back to the innervating axon terminal and activates presynaptic programs necessary for synaptic growth. Thus, increased postsynaptic markers in SCA7 likely send a retrograde signal to elevate presynaptic activity in SCA7 NMJs.

During muscle regeneration, an inflammatory response is triggered that includes the chemotaxis of growth factors, cytokine production by macrophages and fibroblasts, with activation and proliferation of satellite cells. These events differentiate myoblasts into myocytes that fuse to form myofibers, leading to centralization of nuclei (47, 48, 83). S100β is released from impaired myofibers and satellite cells, which attracts macrophages and promotes their polarization into an M2 (pro-regenerative) phenotype (84). Muscles overexpress proteins such as TGF-β1 during muscle regeneration, which promotes the formation of fibrotic tissue (51). In SCA7 respiratory muscles, we found that the diaphragm was intact, while the tongue was impaired. We found increased centralization of nuclei in the genioglossal muscle of the tongue along with increased expression of *Tgfb1*, which indicates fibrosis. Similarly, we found increased *S100b* in SCA7 tongue, which suggests an increase in the pro-regenerative phenotype. These tongue pathologies may also contribute to dysphagia and dysarthria, which are prevalent in SCA7 patients (3, 85). This phenotype can be caused by modulation of signaling cascades influenced by intranuclear accumulation of ataxin-7 in muscle fibers. Another explanation would involve a feed-forward mechanism driven by pathology in XII motor units. Further studies are required to dissect the mechanism of this phenotype and also understand why is it differentially regulated in different types of muscles in SCA7 mice.

### Conclusion.

In conclusion, we demonstrate significant pathology in the XII and putative phrenic motor nuclei, nerves, and NMJs that are important centers for control of the upper airways and for diaphragm function. This pathology explains the dysphagia, aspiration, and respiratory pathology that afflict infants with SCA7. Furthermore, we confirm the role of astrocytes and oligodendrocytes in neuronal and nerve pathology and the importance of neuronal-glial interactions in SCA7. These findings underscore the significance of respiratory pathology in SCA7 and the importance of targeting the respiratory centers with future therapies.

## Methods

### Sex as a biological variable.

We did not identify sex differences in our experiments; therefore, both male and female were equally represented in our experiments.

### Mice.

SCA7^266Q/5Q^-knockin mice (*Mus musculus*) were generated by the Huda Zoghbi lab (Baylor College of Medicine, Houston, Texas, USA) (20). All experiments were performed with SCA7^266Q/5Q^-knockin and WT mouse littermates. WT and SCA7^266Q/5Q^ males and females were analyzed unless indicated otherwise. Animals were bred and housed at the Duke University Division of Laboratory Animal Resources on a 12-hour light/dark cycle with ad libitum access to food and water. These mice were provided chow and HydroGel (clear H_2_O) packs to supplement their regular food and water supply from 8 weeks of age. Mice were euthanized at a predetermined humane endpoint.

### Immunohistochemistry.

Mice were anesthetized with inhaled isoflurane and euthanized via double thoracotomy followed by removal of the heart. Spinal cords and brainstems were harvested along with vertebrae and lower cranium, and postfixed for 48–72 hours in 4% paraformaldehyde (PFA). The spinal cord and brain stem were extracted from the surrounding soft tissue and bone, placed in 4% PFA for 24 hours, and then transferred to 30% sucrose. The cervical spinal cords and medullas were embedded in TissueTek optimal cutting temperature compound (Sakura Finetek, 4583), cut in cross sections (20 μm) using a Leica CM3050 S cryostat, and stored in 2% PFA. Free-floating sections were washed in PBS, quenched with hydrogen peroxide for 1 hour, blocked with 10% normal horse serum for 1 hour (Vector Laboratories, S-2000), and incubated overnight with anti-ChAT (1:250; Millipore, AB144P) or anti-GFAP (1:500; nCor Biotechnology, CPCA-GFAP, AB_2109953), and anti–ataxin-7 (1:500, Invitrogen, PA1-749). Subsequently, the tissues were incubated with anti–goat IgG Alexa Fluor 488 (1:500; Invitrogen, A32814) or anti–chicken IgY Alexa Fluor 647 (1:500; Invitrogen, A21449) and anti–rabbit IgG Alexa Fluor 594 (1:500; Invitrogen, A32754). Sections were mounted in Vectashield Antifade Mounting Medium with DAPI (Vector Laboratories). For negative control staining, tissues were stained with secondary antibodies only. For TUNEL staining, the PFA-fixed tissues were washed, blocked, and stained as described above. These tissues were subsequently treated with 50 μL of TUNEL Enzyme Solution and 450 μL Label Solution, as per the manufacturer’s protocol (In Situ Cell Death TMR, Roche, 12156792910). Then, they were mounted in Vectashield Antifade Mounting Medium with DAPI. Images were acquired using a Zeiss 780 upright confocal microscope and analyzed using Zen 3.3 Blue acquisition software (Zeiss Inc.) Maximum projection images from confocal *Z*-stacks were acquired. Care was taken to minimize pixel saturation while imaging each *Z*-stack. No fluorescence crossover was found between the channel and images were collected separately using appropriate laser excitation. Three to 4 animals per genotype were examined and representative images are shown. Putative phrenic and XII motor pools and motor neurons were identified in each section based on size, morphology, and location, and quantified as previously described (86–88).

To quantify astrocytes, total numbers of DAPI^+^ cells as well as DAPI^+^GFAP^+^ double-positive cells were counted in XII and phrenic respiratory control centers from 4 animals per genotype. The data are presented as total percentage of DAPI^+^GFAP^+^ double-positive cells. Analysis was performed by blinded observers using ImageJ (NIH).

### NMJ staining.

The procedure is adapted from a previously described protocol (89). Whole-mount diaphragms were harvested from 9-week WT and SCA7 mice. The diaphragms were finely teased in 1× PBS and then fixed in 2% PFA for 15 minutes followed by washes with PBS. The tissues were stored at 4°C for 48–72 hours. Diaphragms were permeabilized with 2% Triton X-100 and then blocked overnight at 4°C in blocking buffer (4% normal horse serum, 2% BSA, 0.5% Triton X-100 in 1× PBS) followed by incubation with anti-ZNP1 (1:200; Zebrafish International Research Center, znp-1 090811) and anti–NF-H (1:1200; EnCor Biotechnology Inc., CPCA-NF-H) at 4°C for 48 hours, washed with PBS, and incubated with secondary antibody solution consisting of anti-mouse Alexa Fluor 488 (1:200; Invitrogen, A11001), anti-chicken Alexa Fluor 647 (1:1200; Invitrogen, A21449), and Alexa Fluor 594–conjugated α-bungarotoxin (1:1000; Invitrogen, B13423) for 2 hours at room temperature. The tissues were mounted with Fluoro-gel with Antifade and Fluoro-Gel with Tris Buffer (Electron Microscopy Sciences, 17985) and sealed with clear nail polish. Images were acquired from 5 different fields randomly selected from each mouse diaphragm using a Zeiss 780 upright confocal microscope at ×20 magnification. Maximum projection images from confocal *Z*-stacks were acquired. All quantifications were performed using ImageJ by 2 researchers blinded to genotype. The number of NMJs from each field was quantified using ImageJ and an average of number of NMJ clusters in each diaphragm is reported. Colocalization between individual presynapses and their endplates was calculated using Fiji’s Coloc 2 plugin and reported as a Pearson’s correlation coefficient ranging from –1 (perfect anti-correlation) to 1 (perfect correlation). Endplate areas were calculated using Fiji’s measurement tool.

### Nerve processing and imaging.

Phrenic and XII nerves were harvested from 9-week WT (*n* = 4) and SCA7 mice (*n* = 4). Nerves were placed in 2.5% glutaraldehyde and 0.1% sodium cacodylate. They were then processed, embedded in hard plastic, sectioned to 1 μm, and stained with 1% toluidine blue and 1% sodium borate by the Duke University Electron Microscopy Core. Semithin sections were imaged in brightfield using an ECHO Revolve microscope. Light micrographs were analyzed using the public downloadable ImageJ gRatio plugin to examine the g-ratio, fiber diameter, axon diameter, and myelin thickness. The g-ratio is the measure of the ratio of the axon diameter to the diameter of the axon plus myelin, and is a highly reliable indicator of optimal myelination. One hundred randomly selected axons from each nerve were manually outlined for each animal.

For electron microscopy, phrenic and XII nerves from WT (*n* = 4) and SCA7 (*n* = 4) mice were placed in 2.5% glutaraldehyde and 0.1% sodium cacodylate and then postfixed in 1% osmium tetroxide. The nerves were placed in 1% uranyl acetate and then dehydrated with acetone. They were then processed in epoxy resin (EPON), cut into 60-nm ultrathin sections on a Reichert Ultracut E ultramicrotome, and stained with 2% uranyl acetate and SATO’s lead stain. The nerves were imaged on a Philips CM12 electron microscope.

### qPCR.

Total RNA was prepared using TRIzol (Life Technologies) from flash-frozen tissues (tongue, diaphragm, medulla, and cervical spinal cord). One microgram of RNA was reverse transcribed using the high-capacity cDNA Archive kit (Applied Biosystems). Real-time qPCR was performed using TaqMan Gene expression Master Mix (Applied Biosystems) and TaqMan primers to evaluate gene expression. Gene expression levels of the target genes were normalized to housekeeping gene *Gapdh* and are presented as fold change over control. Data are presented as mean ± SEM of biological repeats.

### Neurophysiology.

The nerve recording procedure was adapted as previously described (90). Nine-week WT (*n* = 4) and SCA7 (*n* = 7) mice were anesthetized by intraperitoneal urethane (1.5 g/kg; Sigma-Aldrich) and placed supine. Body temperature was maintained at 37.3°C to 37.4°C using a servo-controlled heating pad (model TC-1000, CWE). Pulse oximetry (MouseOx, Starr Life Sciences) was used to measure the saturation of hemoglobin (O_2_ saturation [Sa_O2_] as percentage). The trachea was cannulated below the larynx to enable mechanical ventilation (MicroVent, Harvard Apparatus) with a hyperoxic gas mixture (inspired O_2_ fraction [Fi_O2_] = 0.50]). End-tidal CO_2_ (ET_CO2_) was monitored (MicroCapStar, CWE) and correlated with an arterial blood gas (ABL80 Flex, Radiometer America Inc.), with the overall goal of maintaining a PCO2 of approximately 40 mmHg. During this period, the limb-withdrawal response to toe pinch was monitored to ensure the adequacy of anesthesia, and supplemental urethane was given if indicated (0.3 g/kg i.p.). After initiating mechanical ventilation, muscular paralysis was induced with pancuronium bromide (2.5 mg/kg i.p.; Sigma-Aldrich) to eliminate respiratory muscle contraction. Mice were bilaterally vagotomized to prevent entrainment of XII motor output with the ventilator. The right XII nerve was isolated at the distal end. Nerve activity was recorded using a bipolar suction electrode (A-M Systems, 573040) following amplification (1,000×; Model 1700, A-M Systems). Neurograms were integrated using a 100-ms time constant (model MA-821, CWE). Data were digitized using a CED Power 1401 data acquisition interface and recorded on a personal computer using Spike2 v8.21 software (Cambridge Electronic Design). Baseline recordings were determined by an even amplitude and stable ET_CO2_. Five minutes of baseline recordings were obtained and then followed by a 10-minute hypercapnia challenge (Fi_O2_ 0.21; Fi_CO2_ 0.07; nitrogen balance) recording. Respiratory rate and tidal volume were the same at baseline and challenge. An arterial blood sample was obtained for measurements of arterial blood gas in a subset of mice (ABL80 Flex, Radiometer America Inc.). Mice were then euthanized after the procedure by secondary organ removal. The percentage of baseline was calculated as (challenge – baseline)/baseline × 100.

### RNA-seq.

The medulla and cervical spinal cord tissues from P8, 5-week, and 9-week SCA7 (*n* = 9) and WT mice (*n* = 8) were harvested and flash frozen. RNA was extracted using a Qiagen miRNeasy kit. Initial processing and analysis of sequencing results were performed by Duke Genomic Analysis and the core facility. Reads were mapped to the GRCm38v73 version of the mouse genome. Reads were kept for subsequent analysis if they mapped to a single genomic location. Only genes that had at least 10 reads in any given library were used in subsequent analysis. Normalization and differential expression were carried out using DESeq2 (https://www.bioconductor.org/packages/release/bioc/vignettes/DESeq2/inst/doc/DESeq2.html). The false discovery rate (FDR) was calculated to control for multiple hypothesis testing. Upregulated and downregulated genes were analyzed based on log_2_(fold change).

For GO enrichment analysis, overrepresentation analysis was achieved using the Clusterprofiler package (https://bioconductor.org/packages/release/bioc/html/clusterProfiler.html). Samples were first grouped according to their genotype, age, and tissues. For each group, genes that significantly differed in expression (*P* < 0.01) were selected for further analysis. Fisher’s exact test was used to determine whether there was more overlap between the differentially expressed gene list and the GO annotation list than would be expected by chance.

### Statistics.

Statistical analyses were performed using GraphPad Prism 9 using a 2-tailed Student’s *t* test between 2 genotypes. Further information is indicated in the figure legends. For all statistical analyses, significance is defined as a *P* value of less than 0.05: *P < 0.05, **P < 0.01, ***P < 0.001, ****P < 0.0001. All data are presented as mean ± SEM.

### Study approval.

Animal care and all experimental procedures included in this study were approved by the Institutional Animal Care and Use Committee of Duke University.

### Data availability.

The values for all data points shown in graphs are provided in the Supporting Data Values Excel file. The custom code is available through GitHub (https://github.com/MaiElMallah/RNA-Seq_SCA7_medulla_cervical).

The RNA-seq data are available in the NCBI Gene Expression Omnibus (GEO) (accession number GSE271392; https://www.ncbi.nlm.nih.gov/geo/query/acc.cgi?acc=GSE271392).

## Author contributions

DDB conceptualized the study, curated data, analyzed data, conducted experiments, developed methods, provided project administration, validated data, generated figures, wrote the original manuscript draft, and reviewed and edited the manuscript. YS, LEH, RS, LMS, LAP, ERS, and AH curated data, analyzed data, conducted experiments, generated figures, and reviewed and edited the manuscript. RS, JSD, MH, and SK analyzed data, conducted experiments, generated figures, and reviewed and edited the manuscript. ARLS provided resources and reviewed and edited the manuscript. MKE conceptualized the study, analyzed data, acquired funding, conducted experiments, developed methods, provided project administration, resources, and supervision, and reviewed and edited the manuscript.

## Supplementary Material

Supplemental data

Supporting data values

## Figures and Tables

**Table 2:**
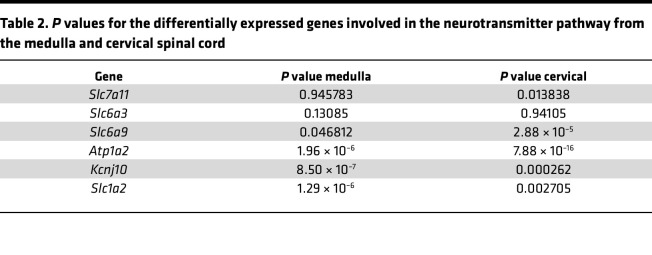
*P* values for the differentially expressed genes involved in the neurotransmitter pathway from the medulla and cervical spinal cord

**Table 1:**
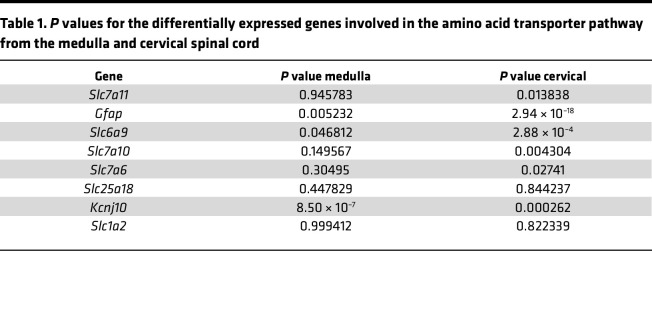
*P* values for the differentially expressed genes involved in the amino acid transporter pathway from the medulla and cervical spinal cord

**Table 3:**
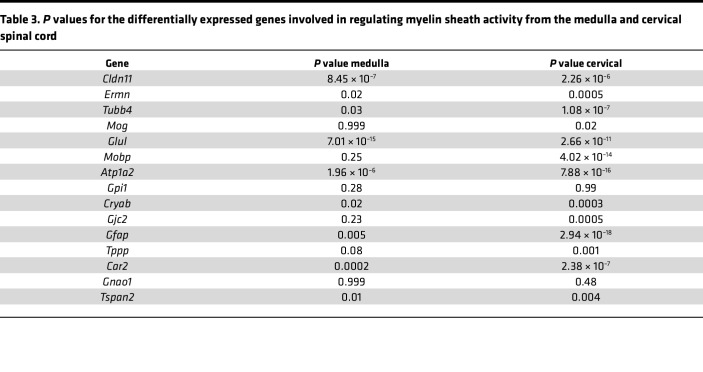
*P* values for the differentially expressed genes involved in regulating myelin sheath activity from the medulla and cervical spinal cord

## Version 1 07/25/2024

In-Press Preview

## Version 2 09/24/2024

Electronic publication
