# Supplementary material for: Respiratory neuropathology in spinocerebellar ataxia type 7
Source: JCI Insight. 2024 Jul 18;9(18):e170444. doi: 10.1172/jci.insight.170444 (PMC11457860; doi:10.1172/jci.insight.170444)
Supplement: Supplemental data [file jciinsight-9-170444-s212.pdf]

Supplementary figures:

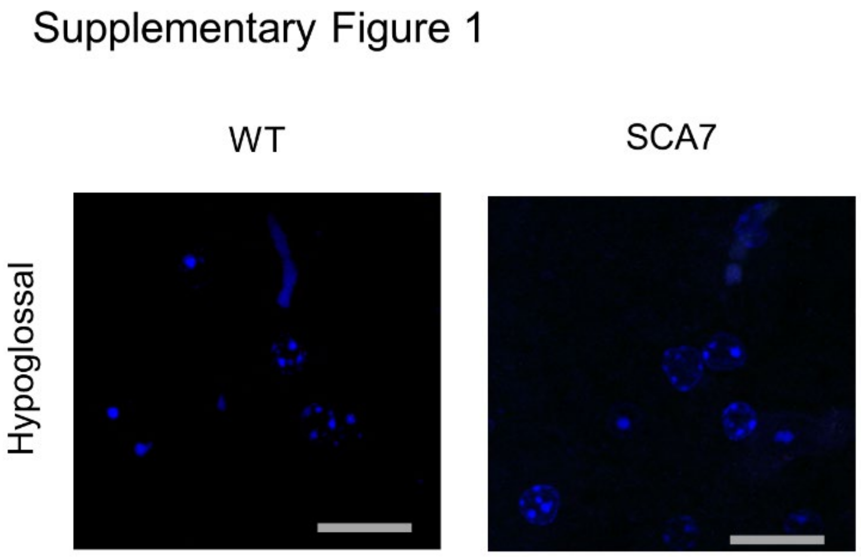

Supplementary Figure 1: Representative confocal images from 9-week WT (A) and SCA7 (B) XII respiratory center immunostained for secondary antibody anti-chicken IgY (secondary antibody for GFAP) and anti-rabbit IgG Alexa Fluor 594 (secondary antibody for ataxin-7). Cell nuclei were visualized by DAPI (blue). Scale bars represent 20 μm.

Supplementary Figure 2

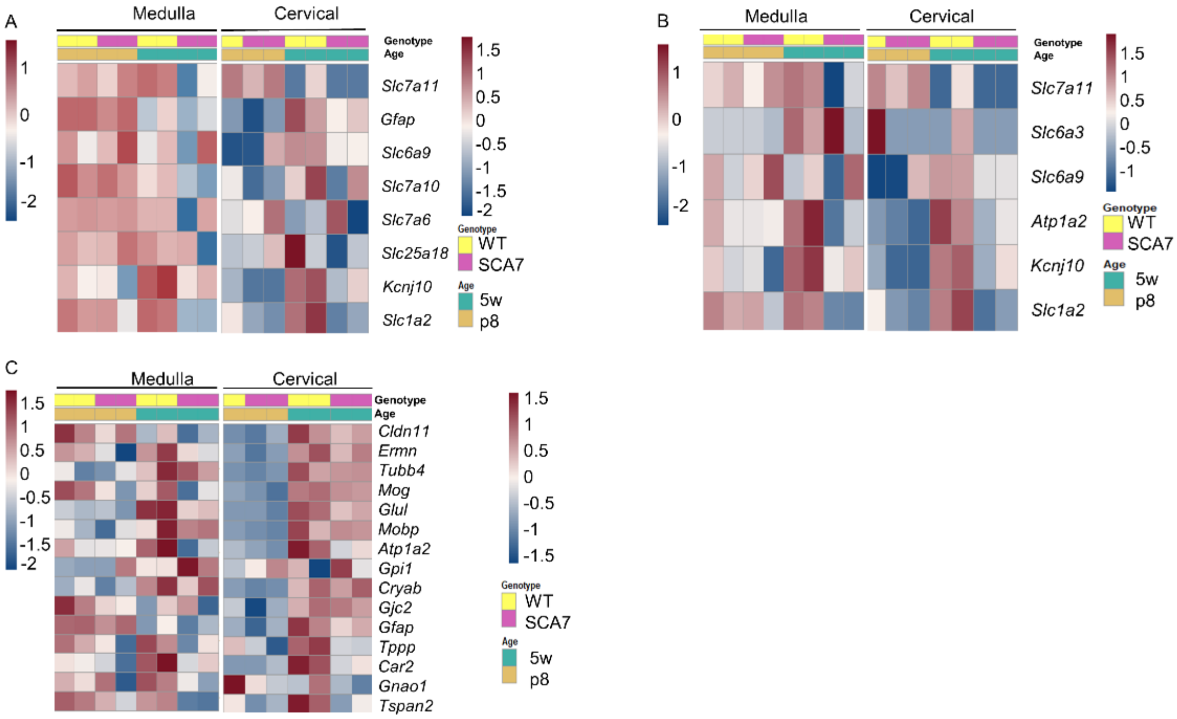

Supplementary Figure 2: Transcriptome profile of proteins involved in amino acid transmembrane transportation (A) and neurotransmitter transport (B) myelin sheath (C) activity pathways of 8-day (p8) and 5-week WT and SCA7 mice. (n=2 per genotype and age group).

Supplementary Figure 3

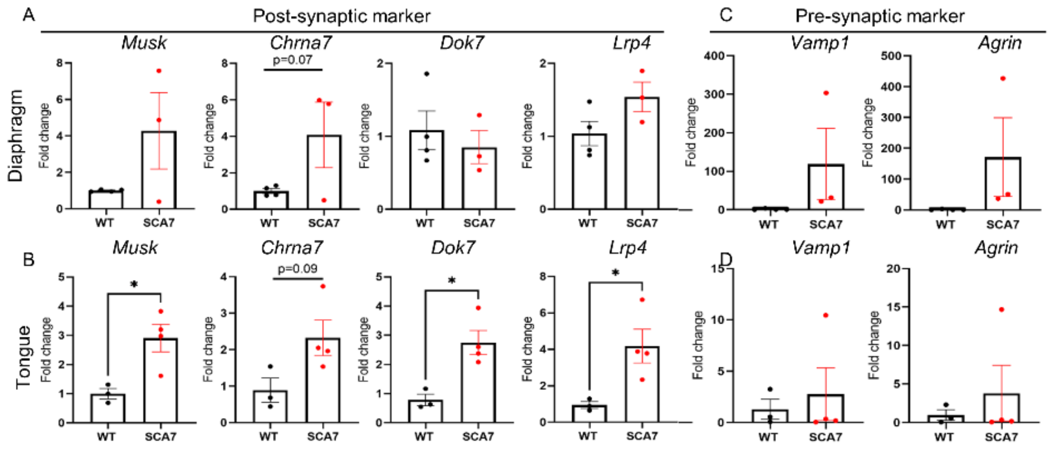

Supplementary Figure 3: (A-D) Expression of postsynaptic markers (A, B) and presynaptic markers (C, D) in the diaphragm (A, C) of 5-week WT (n=4) and SCA7 (n=3) mice and tongue (B, D) of 5-week WT (n=3) and SCA7 (n=4) mice. *p<0.05, Student's *t*-test.

Supplementary Table 1: Fold change in WT Vs SCA7 medulla at 9 weeks of age

DE genes	log2FoldChange	P value
Glul	1.426108372	7.01E-15
Apod	-1.114248715	1.31E-07
Fa2h	1.955504924	1.31E-07
Kcnj10	1.477889734	8.50E-07
Cldn11	1.424775037	8.50E-07
Ndrg2	1.429307468	1.06E-06
Cdkn1a	-3.19639236	1.06E-06
Ugt8a	2.398636517	1.06E-06
Trf	1.22226936	1.20E-06
Slc1a2	2.24880082	1.29E-06
Atp1a2	1.2201349	1.96E-06
Gja1	1.414172117	1.96E-06
Plpp3	1.490730149	1.44E-05
Plp1	1.679566843	1.61E-05
Qdpr	1.183321137	2.06E-05
Scd2	1.233934461	3.29E-05
Atp1b2	1.303460325	4.53E-05
Ttyh1	1.119129239	0.000155
Gpr37l1	1.249436305	0.000176
Rbm3	-1.726213182	0.000176
Car2	1.630938721	0.000201
Gpr17	2.171718793	0.000231
Maff	-3.666265257	0.000248
Kcnk13	2.788477799	0.000252
Polr2a	-1.222364914	0.000313

Mmd2	1.472658369	0.000407
Agt	1.633143228	0.000447
Itih3	1.482232474	0.000459
Aqp4	1.247921682	0.000459
Fkbp5	-1.766593614	0.000595
Il33	2.48400154	0.00208
Btbd17	2.335732961	0.002565

Supplementary Table 2: Fold change in WT Vs SCA7 cervical spinl cord at 9 weeks of age

DE genes	log2FoldChange	P value
Gfap	2.287255	2.94E-18
Plp1	2.55993	2.94E-18
Atp1a2	1.725704	7.88E-16
Mobp	2.309787	4.02E-14
Ndrg2	1.712322	4.07E-12
Glul	1.391906	2.66E-11
Prr18	2.610351	1.22E-10
Myoc	3.7587	3.57E-10
Ugt8a	2.435472	1.19E-09
Agt	2.158251	4.62E-09
Il33	2.42421	3.23E-08
Fa2h	1.961813	4.47E-08
Tubb4a	1.236467	1.08E-07
Car2	1.677331	2.38E-07
Padi2	1.955912	2.41E-07
Qdpr	1.998996	2.41E-07
Cldn11	1.352844	2.26E-06
Trf	1.382898	2.68E-06
Aebp1	2.487037	2.76E-06
Klk6	2.48684	2.76E-06
Igfbp5	2.603111	5.12E-06
Prg4	5.992226	5.88E-06
Serpind1	4.623417	5.88E-06

Hbb-bs	2.915133	1.13E-05
Fn1	2.484543	1.83E-05
Nnat	2.409432	1.95E-05
Slc6a9	1.790332	2.88E-05
ND2	1.860178	3.78E-05
C3	2.067771	4.40E-05
Tuba1a	1.05779	7.78E-05
Igf2	4.444373	7.78E-05
Desi1	1.500739	8.87E-05
Wnk1	1.143189	0.000109
Neat1	1.302376	0.000114
Dbp	1.772665	0.000118
Trp53inp2	1.505447	0.000137
Cst3	1.314907	0.000181
Slain1	1.146795	0.04544
H1fx	1.569985	0.047231
Slco3a1	1.207556	0.047866
Rab7b	3.270351	0.048752
Btbd17	2.988751	0.048752
Nes	2.359896	0.048956
Mt2	1.139638	0.049327
Cxcl12	1.98618	0.001572
Slc13a4	4.611446	0.001612
Aldh1a2	3.77626	0.001713
Mal	1.072664	0.001738
Pltp	2.421986	0.001853
Fth1	1.496091	0.001903

Gjc3	1.152565	0.00197
Fxyd1	1.926078	0.002079
B230206H07Rik	3.311574	0.002247
Metrn	1.612594	0.002575
Hcn2	0.941991	0.002705
Rida	1.729324	0.002705
Slc1a2	1.742119	0.002705
Mpz	6.561351	0.002794
Kif13b	1.411272	0.002864
Islr	3.646104	0.002888
Tmem88b	1.022393	0.002966
Unc5b	1.601644	0.002977
Gamt	2.813142	0.003236
Aldh1a1	1.202588	0.003315
Gltp	1.437841	0.003659
Aqp4	1.411911	0.003688
Tspan2	1.117648	0.003785
Scd1	1.002314	0.004108
Itih3	1.3762	0.004304
Slc7a10	1.451063	0.004304
Slc7a11	2.90732	0.013838
Hsph1	1.1316	0.014223
Clec3b	2.902872	0.01424
Fam129b	1.588886	0.014455
Kcna6	1.580202	0.015664
Lgi4	1.676615	0.01663
Slc25a1	1.236293	0.017224

Cilp	4.373834	0.017819
Atp1b2	0.956684	0.018589
Fads1	1.239999	0.018589
Col3a1	3.315298	0.018994
Crispld2	2.769331	0.018994
Mfap5	4.40678	0.019848
Kdm3a	-1.35412	0.020619
Msmo1	1.035128	0.020619
Itpkb	1.067801	0.020711
Gpr37l1	0.990862	0.004334
Fmod	3.11875	0.001384
Col1a1	2.940252	0.001384
Ntsr2	1.780519	0.001384
Tppp	1.229707	0.001433
Bgn	2.324043	0.001443
Nbl1	2.189804	0.001559
Sema3b	1.785205	0.021678
Dao	2.552329	0.021703
Hspg2	1.820137	0.0237
Gm39080	3.698217	0.0237
Mog	1.125914	0.023902
Ptn	1.470051	0.024102
Mbp	1.466421	0.024542
Atp5d	1.055038	0.025971
Vwa1	1.383213	0.025981
Aplnr	3.36666	0.027426
Sfxn5	1.027763	0.027674

Rhog	1.399937	0.02833
Ndrg1	1.248584	0.029534
Gpr17	2.142677	0.029865
Prelp	2.334158	0.030993
Calr	0.884732	0.030993
Pllp	0.997847	0.031302
Tpt1	1.0637	0.031392
Rbp4	3.06672	0.032517
Clic4	1.002389	0.033139
Gareml	1.820883	0.033139
9430020K01Rik	1.466295	0.033266
Arhgef19	1.655063	0.033303
Ralgps1	-1.01956	0.033836
H19	4.279946	0.033836
Paqr8	1.096627	0.033938
S100b	1.008759	0.033938
Dock5	1.190733	0.033938
Sox2ot	1.077408	0.034492
Mgp	4.206475	0.035479
ND1	1.304199	0.03639
Hbb-bt	3.114968	0.038272
Slc22a6	4.440406	0.039842
CYTB	1.353466	0.043365
Epb41l2	1.18523	0.043465
Gpr37l1	0.990862	0.004334
Ttyh1	0.981799	0.004334
Mmd2	1.563144	0.0048

Mt3	1.416983	0.005516
Hif3a	-1.57702	0.005742
Lcat	2.126896	0.005742
C4b	1.751635	0.005757
Enho	1.341414	0.006928
Ppp1r14a	1.656496	0.007704
Gja1	1.080018	0.008255
Mag	0.860377	0.008804
Lpar1	1.185601	0.010287
Efhd1	1.357831	0.010472
Zkscan16	-1.34061	0.010472
Ahnak	1.317273	0.010493
Abca1	-1.4652	0.010755
Tmem229a	1.192775	0.010755
Tet2	-1.58672	0.01081
Wnt4	3.684656	0.01093
Slc22a8	2.131949	0.011033
Gatm	0.983194	0.011126
Mtss1l	1.105475	0.011278
Kcnk13	2.292378	0.01204
Micall1	1.129448	0.01204
Cirbp	-1.06889	0.012404
Panx2	1.264029	0.012691
Scd2	1.120291	0.012691
Heyl	2.573085	0.012792
Ogn	3.733808	0.013345
Mapt	0.990768	0.013412

Lyz2	2.931286	0.013767
Sox10	1.03777	0.013767
Igfbp6	5.034083	0.020711
Podxl	1.836262	0.021416
